# Bacterial Abundance and Physicochemical Characteristics of Water and Sediment Associated with Hydroelectric Dam on the Lancang River China

**DOI:** 10.3390/ijerph16112031

**Published:** 2019-06-07

**Authors:** Xia Luo, Xinyi Xiang, Guoyi Huang, Xiaorui Song, Peijia Wang, Kaidao Fu

**Affiliations:** 1 Institute of International Rivers and Eco-Security, Yunnan University, Kunming 650500, China; luoxia@ynu.edu.cn (X.L.); xiangxy1994@126.com (X.X.); 18469154604@163.com (G.H.); huangtinshu@foxmail.com (X.S.); wangpeijia19@163.com (P.W.); 2 Yunnan Key Laboratory of International Rivers and Transboundary Eco-Security, Kunming 650500, China

**Keywords:** bacterial abundance, physicochemical characteristics, hydroelectric dam, water and sediment, seasonality

## Abstract

Research on bacterial abundance in water column and sediment of dammed rivers remain poorly understood, despite their importance to biogeochemical processes, benthic ecology, and bioremediation. The present study investigates the water and sediment bacteria by epifluorescence microscopy in the reservoir (above the dam site), as well as in the downstream river stretches (below-dam site) at the middle reach of Lancang River during the wet, the normal and the dry seasons. The results demonstrated that the reservoir operating regime (water discharge variations) and strong precipitation promoted significant differences in the conditions of the river below the dam, especially for the concentration of dissolved oxygen, redox potential, electric conductivity, turbidity, and total dissolved solids in water and concentration of microbial activity in sediment. The seasonal variations were also key factors influencing water quality at the below-dam sampling sites. Nutrients concentration did not induce a significant response in bacterial abundance when inorganic nutrients were sufficient. Bacterial density in sediment was regulated by hydropower-related discharge, particle size, and type of sediments, while bacterial abundances in water were strongly linked with the physicochemical characteristics of the water, such as total dissolved solids and conductivity.

## 1. Introduction

As one of the most important components in an aquatic ecosystem, bacteria have been found almost anywhere water exists, and its spatial and temporal distribution is closely tied to water quality and possible presence of pathogens [1]. It is now established that bacterial abundances in both freshwater and marine worldwide play a key role in elemental biogeochemical cycles. In addition to their biogeochemical impacts, bacterial abundance may have significant ecological impacts upon in situ bioremediation in both freshwater sediments and waters [2,3]. Therefore, bacterial abundance has been widely studied in marine and limnetic waters. Investigations have suggested that bacterial abundance increases with the trophic state of freshwater [4]. Weak but significant positive correlations between bacterial numbers and both total nitrogen (TN) and total phosphorus (TP) have also been reported elsewhere [5,6]. Studies of aquatic food webs have found bacterial abundance, chlorophyll a (Chl a) concentrations, and temperature have been linked to both bacterial production and specific growth rates [7,8,9], and hence bacterial abundance has also been shown to be a good predictor of bacterial production in freshwater systems [10]. 

Bacteria are 2–1000 times more abundant in sediment than in the overlying water column of freshwater ecosystems [11], and thus bacteria in sediments have potential to influence the chemistry of the overlying water through the reduction of nitrate, sulphate, and methane [12], the release of phosphorus [13], and ammonium from sediments [13,14], as well as bacterial consumption of hypolimnetic oxygen [15].

Our understanding of the interactions and relationship between bacterial abundance and nutrient balance due to river damming is still limited [16]. Previous studies showed that the damming of rivers, as one of the primary man-induced disturbances, can greatly alter river hydrology, reservoir storage volume due to sedimentation [17], aquatic ecosystem upstream and downstream of the dam [18], riverborne nutrient loads [19], and oxygen and thermal conditions [20]. All these factors dramatically change the water–sediment exchanges and the proportions of bacteria in water and sediment of dammed reservoir; for example, construction of dams in rivers may cause considerable reductions in nutrient loads as a result of the removal of nitrogen and phosphorus in reservoir sediments [19]. However, the effect this may have on bacterial density and trophic conditions (measured as the concentration of Chl a) is largely unknown.

The Upper Lancang–Mekong River, also called the Lancang River in China, is of great importance, not only to Southwestern China, but also to the rest of Southeast Asia. In recent decades, the Lancang River Basin has experienced an increase in anthropogenic disturbances, such as the construction of hydropower stations. Changes in river continuum and the hydrological conditions due to dam construction can alter biogeochemical nutrient distribution, resulting in water quality problems. In consequence, it is crucial to know the amount of bacteria in the Lancang River, since bacteria not only are favored as decomposers to decompose the high organic load of eutrophic river, but also change inorganic nutrients and dissolved organic substrates into higher trophic levels via predation by protozoan and metazoan plankton. In this study, we investigated the response of bacterial density (by the direct-count method using a fluorescent dye and an epifluorescence microscope) to impoundments by river damming. Furthermore, we have also analyzed the effects of hydropower-related discharge on physicochemical characteristics and further on the bacterial abundance in present study. Important ecological questions arose from the above-mentioned studies on the portion of freshwater bacteria about the environmental factors inhibiting or limiting bacterial abundance: (1) How would the bacterial abundance change in response to impoundments by river damming? Are changes in bacterial abundance by river damming driven actually by the flow of nutrients, in particular TP and TN? (2) Does hydropower-related discharge affect water quality and further to affect the bacterial density of dammed reservoir and downstream river? Does it influence also stratification in the dammed reservoir? (3) How important is temperature as a regulating factor for bacterial density under the influence of dam-induced modifications? 

## 2. Materials and Methods

### 2.1. Study Site and Sampling 

**Figure 1:**
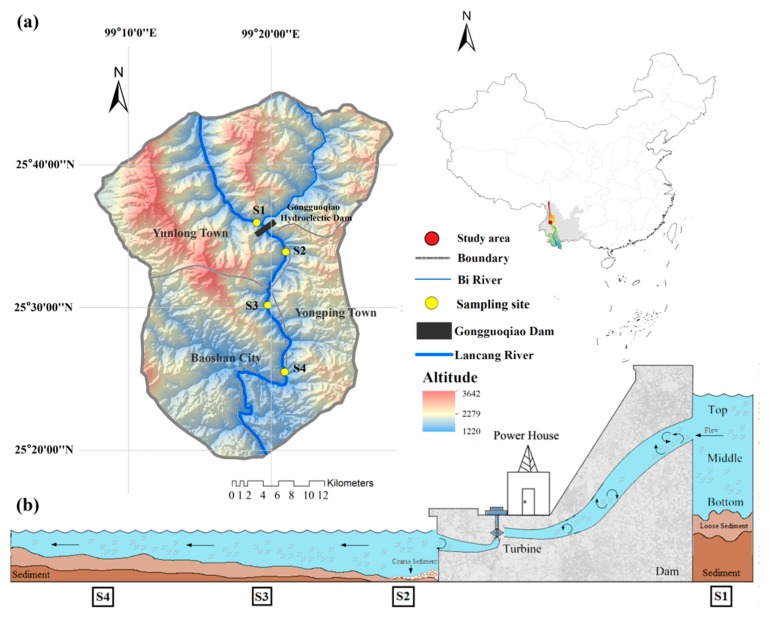
Map of the sampling locations. (**a**) Location of sampling sites in the upstream and downstream of Gongguoqiao hydroelectric dam in the Lancang River Basin, Yunnan Province, China. (**b**) Schematic showing the dam and four sampling stations.

This study was conducted on the upstream and downstream riverine zones of Gongguoqiao hydroelectric dam (Figure 1). This hydroelectric dam is located in the middle reach of the Lancang River with a height of 10^5^ m, and is the first stage of the middle reach of Lancang mainstream cascade development project. The reservoir area is 155,400 km^2^ with a backwater of 46 km, and the total reservoir capacity is 3.6 × 10^8^ m³ with an annual average flow of 1010 m^3^/s (see Table S1 in Supplemental Materials for more details). 

In this study, four sampling sites were chosen to investigate the impact of impoundments due to river damming on bacterial abundance and physiochemical characteristics of water and sediment. Site 1 (S1) was located approximately 0.5 km upstream of the dam, site 2 (S2) was situated just downstream of the dam, and site 3 (S3) and site 4 (S4) were situated approximately 7 km and 15 km after S2, respectively. S4 was supposed to locate just upstream of the Xiaowan Reservoir (i.e., the second reservoir in the cascade dams). Surface water samples (20 cm—the most common depth for bacterial sampling) [21,22] and surface sediment (10 cm) samples were collected in dry- (January), normal- (May) and wet seasons (July), 2017 from S1 to S4 along the Lancang River, among which water samples from the top, the middle, and the bottom of S1 were also taken for bacterial abundance, Chl a, and water quality analysis to assess the influence of hydropower-related discharge on reservoir stratification (see Table S2 in Supplemental Materials). 

On each sampling date, triplicate water samples were collected at each site in three sterile 500 mL polythene bottles and stored at 4 °C in dark for nutrient analyses, Chl a, and bacterial abundance. Sediment samples at S1 were collected in triplicate with a horizontal sediment sampler (inner diameter: 10.1 cm; tube length: 45 cm) at approximately 33.1 ± 12.67 m of water depth in the river main channel, while clayey bank sediments at S2–S4 were collected in triplicate with a soil core sampler (inner diameter: 38 mm; tube length: 50 cm). Sediment samples were then sealed in sterile plastic bags and transported to the laboratory for bacterial activity (stored in dry ice), bacterial abundance (stored at 4 °C in dark) and nutrient analyses (stored at 4 °C in dark). An multi-parameter water analysis instrument (HORIBA-U52, HORIBA Corporation, Japan) was used to obtain data on temperature, pH, electric conductivity (EC), dissolved oxygen (DO), turbidity, total dissolved solids (TDSs), and oxygen reduction potential (ORP) on site. Water and sediment samples were immediately brought to the laboratory and processed as described below.

### 2.2. Water Sample Processing

Each unfiltered water sample was used for analysis of TN and TP. TN was measured by converting all nitrogen forms to nitrate by alkaline persulfate oxidation [23] and subsequent analysis of nitrate by 2,6-dimethylphenol method [24] using a spectrophotometer (UV-5500, Metash Corporation, Shanghai, China). TP was determined by an ascorbic acid method after persulfate digestion [23] using a spectrophotometer (UV-5500, Metash Corporation, Shanghai, China). Chl a was centrifuged at 12,000× *g* for 20 min at 4 °C, followed by acetone extraction [25]. The concentration of Chl a was estimated by optical density measurement at 663, 645, and 630 nm using the SCOR–Unesco equation [26].

Total counts of bacteria in water were performed using acridine orange (AO) according to standard protocols [27]. Briefly, the collected water samples were transferred into sterile test tubes and fixed with 0.2 µm prefilterd formaldehyde to a final concentration of 1% (*v*/*v*). An appropriate amount of water sample (1–5 mL) was filtered onto a black polycarbonate nuclepore filter with a pore size of 0.2 µm and a diameter of 25 mm and stained with the AO at a final concentration of 1 mg·mL^−1^. After staining for 3 min, the water–AO solution was drawn through the filter by suction (125 mm of Hg) and then rinsed with phosphate buffered saline (PBS, pH = 7.4) solution. Bacteria retained on the filters were examined by an Olympus BX51 epifluorescence microscope (Olympus Corporation, Japan) at a magnification of ×1000. On each filter, no fewer than 15 clear-edged cells in 20 randomly selected fields were counted. Total abundance of bacteria was expressed as numbers of bacteria per milliliter of the water sample.

### 2.3. Sediment Sample Processing

Five grams of wet sediment samples from all sites were dried at 105 °C until a constant weight was obtained (approximately 4 h) to determine the sediment water content (WC) [28]. The sediments were reweighed to obtain the oven-dried sediment weight. The dried samples were then placed in a muffle furnace maintained at 550 °C for 5 h to estimate the organic matter (OM) content by the loss on ignition (LOI) method [29]. The OM content was calculated by subtracting the weight of ignited sediment from the weight of oven-dried sediment.

TN and TP in sediment samples were determined by the simultaneous digestion method using potassium peroxodisulfate as an oxidizing reagent [30]. Briefly, 150 mg grinded sediment sample was accurately weighed in a pre-combusted (103 °C, 3 h) corundum crucible and wetted with 100 µL 95% ethanol. Two grams of NaOH were added into the above sample and then subsequently combusted the sample at 720 °C for 15 min. After cooling, the crucible was washed with 100 mL of hot water and filtered (0.45 µm). Seventy milliliters (pH = 6–7) and 30 mL (pH = 3) of the filtrates were digested by the alkaline potassium persulfate solution [31] and then analyzed by a spectrophotometer (UV-5500, Metash Corporation, Shanghai, China) for TP (mg·g^−1^ dry weight (DW)) and TN (mg·g^−1^ DW), respectively. 

The total microbial activity of the sediment samples was measured by the fluorescein diacetate (FDA) hydrolysis technique as described by Schnürer and Rosswall [32]. Briefly, FDA was dissolved in acetone and stored as a stock solution (4.8 mM) at −20 °C. One gram of sediment sample was added into a 150 mL flask filled with 10 mL sterilized PBS (pH = 7.4). 0.5 mL FDA stock was added to start the reaction, and the mixture was incubated at 30 °C for 3 h in dark on a rotary shaker. Once the sample was removed from the shaker, 25 mL of acetone (50%, *v*/*v*) was added immediately to terminate the reaction. The mixture in the flask was shaken vigorously for 30 s and filtered through a 0.45 µm membrane filter to obtain a clear liquid. The filtrates were then measured by a UV-5500 spectrophotometer at an absorbance of 490 nm and calculated using the calibration curve for the total microbial activity (µg FDA·g^−1^ DW·h^−1^).

Additional 5 g of sediment samples from all sites were placed in 150 mL flasks, and treated with 45 mL 1% prefilted (0.2 µm) Tween-80 in PBS (pH = 7.4) for 20 min to detach bacteria from sediment particles. The mixture was shaken vigorously for 5 min and was stayed on the benchtop for another 20 min to separate the solvent from the aqueous phase as described previously by Kepner and Pratt [33]. Approximately 0.2–1 mL of the supernatant was used to estimate the total counts of bacterial cells in the 5 g sediment using the AO-epifluorescence technique as described previously [27].

### 2.4. Statistical Analysis

The repeated measures analysis of variance (ANOVA) was used to analyze each measured physicochemical parameter at different sites over three seasons, as well as the changes in water column chemistry with the reservoir depth (top, middle, and bottom portions of the reservoir) among three seasons. Prior to analyses, the variables were log(x+1)-transformed, centered, and standardized in order to fit the assumption of homogeneity of variance. Significant differences were subsequently tested by post-hoc comparison Tukey’s honestly significant difference (HSD) tests. Mean physicochemical characteristics and bacterial abundance in water and sediment were compared with paired *t*-tests. Pearson correlation was employed to compare all pairs of variables with statistical significance set at *p* < 0.05. Mean bacterial abundance in sediment and water were compared with paired *t*-tests. Statistical analyses described above were performed using the SPSS software (version 20.0) (IBM Corporation, Armonk, NY, USA). Principal component analysis (PCA) in the Canoco software (version 4.5) (Biometris-Plant Research International Wageningen, The Netherlands) program was carried out to ordinate relationships of all the environmental variables at each site. PCA reduces the dimensionality of a large multivariate data set to a smaller number of newly derived orthogonal variables called principal components (PCs) [34,35]. Qualitative data (e.g., geomorphic units) were denoted as dummy variables, whereas quantitative data (e.g., temperature, EC, and turbidity) were log-transformed according to Merovich et al. [34]. The resulting PCA loadings were plotted to evaluate physicochemical properties within and among sites. PCs with eigenvalues of >1.5 were considered significant. Physicochemical characteristics of water and sediment were considered significant components of a PC if their factor loadings had an absolute value of >0.5 as suggested by Hair et al. [36]. The results of the PCA were visualized in the form of an ordination diagram using the CanoDraw tool in Cacono [37,38]. 

## 3. Results

### 3.1. Environmental Variables and Nutrient Concentrations

#### 3.1.1. Changes of General Environmental Parameters 

**Figure 2:**
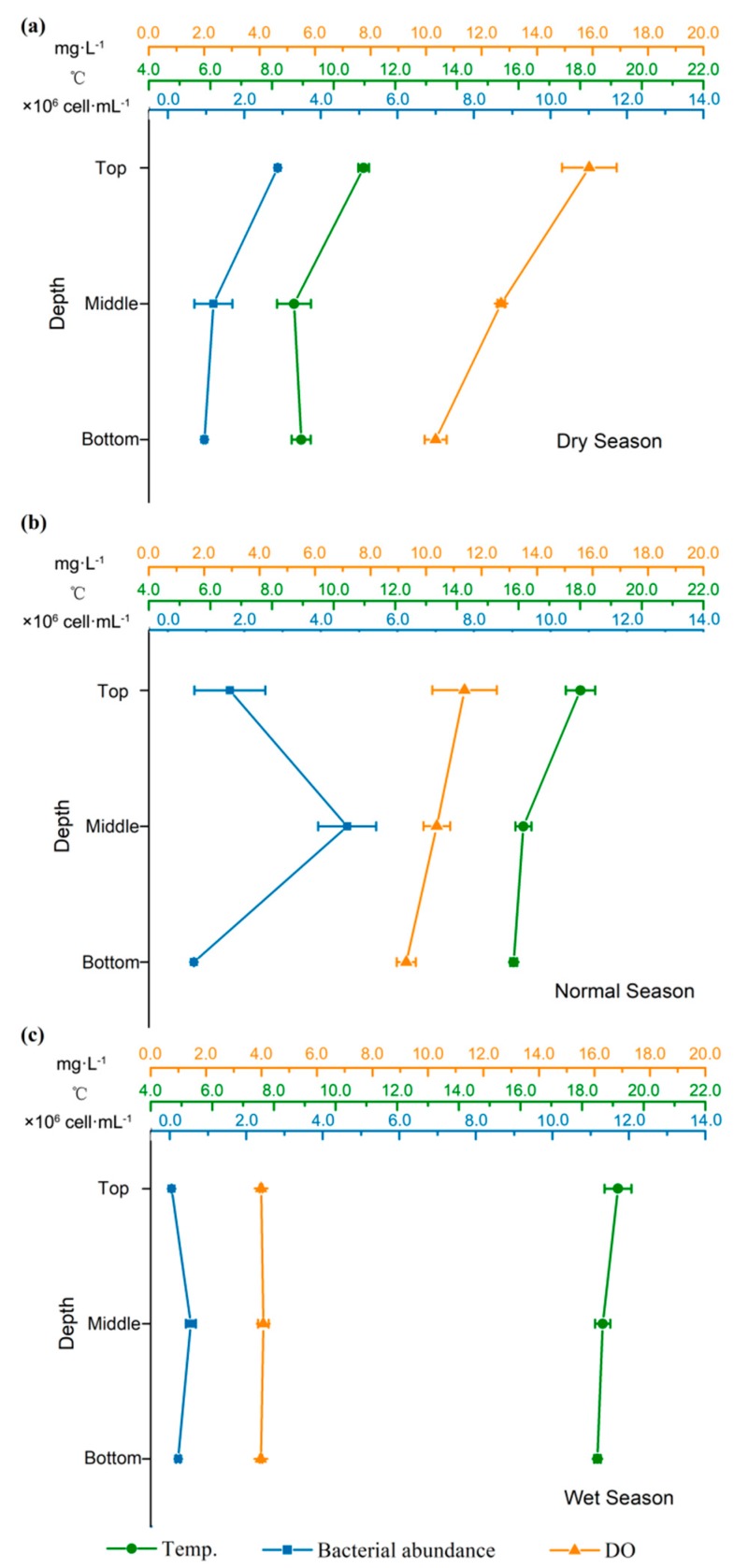
Vertical profile of water temperature, dissolved oxygen concentrations, and bacterial abundance at the Gongguoqiao Reservoir (S1) in dry (**a**), normal (**b**), and wet (**c**) seasons.

Physical and chemical characteristics in surface water and sediment are shown in Table 1 and Table 2, respectively (See Tables S3 and S4 in Supplemental Materials for the raw data). The physicochemical characteristics in both water and sediment exhibited significant spatial and temporal variation (see Table 3). The temperature differed among the sampling times, with a maximum of 20.3 °C and a minimum of 10.2 °C in the rainy and dry seasons, respectively. The water temperature in the dry season (i.e., winter) was vertically homogeneous (only 2 °C difference between surface and bottom) (Figure 2a). The same tendency was observed for the DO in the dry season. In normal and wet seasons, which were characterized by low or intermediate water releases, stratified conditions were not observed in these two seasons (Figure 2b,c). The water temperature below the dam tended to increase during periods of higher water discharge, i.e., the rainy season (Table 1). Precipitation and hydropower-related discharge in the wet season greatly affected physicochemical properties, such as concentration of DO, ORP, EC, turbidity, and TDS in water and WC, OM content, and concentration of microbial activity in sediments. The lowest turbidity (84.9 ± 14.4 nephelometric turbidity units (NTU)) occurred in Gonguoqiao Reservoir (S1) in the dry season, and the highest concentration (10,300.0 ± 667.9 NTU) took place at S4 in winter. There were also elevated and fluctuating turbidity during normal and wet seasons, which were characterized by high discharge and strong precipitation.

Percentages of OM measured in sediment varied significantly (*p* < 0.05) from S1 to S4 over three seasons. Because of enhanced precipitation and discharged occurring in normal and wet seasons, significantly lower microbial activities were recorded than in the dry season.

#### 3.1.2. Changes of TN Concentrations 

For the seasonal sampling periods, there was a significant difference for TN (Table 3). The TN content of surface water at all sampling sites was 3.6 to 6 times greater in the dry season compared with that in the wet season. Low values in the rainy season are directly related to the influence of strong precipitation. In the reservoir (i.e., S1) (see Table S5 in Supplemental Materials), the TN concentrations in different depths differed significantly within the water column in three seasons (*p* < 0.05, Table S6 in Supplemental Materials). There was also a significant difference between TN measured in water and sediment (*p* < 0.05).

#### 3.1.3. Changes of TP Concentrations

In water, TP exhibited significant spatial and temporal differences (Table 3). Higher concentrations of TP were observed in the dry and normal seasons. In the winter, concentrations of TP decreased significantly with depth in the reservoir (S1) (see Table S6 in Supplemental Materials). However, TP concentrations measured in water column were increased gradually in normal and wet seasons at S1 with water depth (Table S5 in Supplemental Materials).

### 3.2. Bacterial Abundance and Algae Biomass

#### 3.2.1. Bacterial Abundances of Water and Sediments

**Figure 3:**
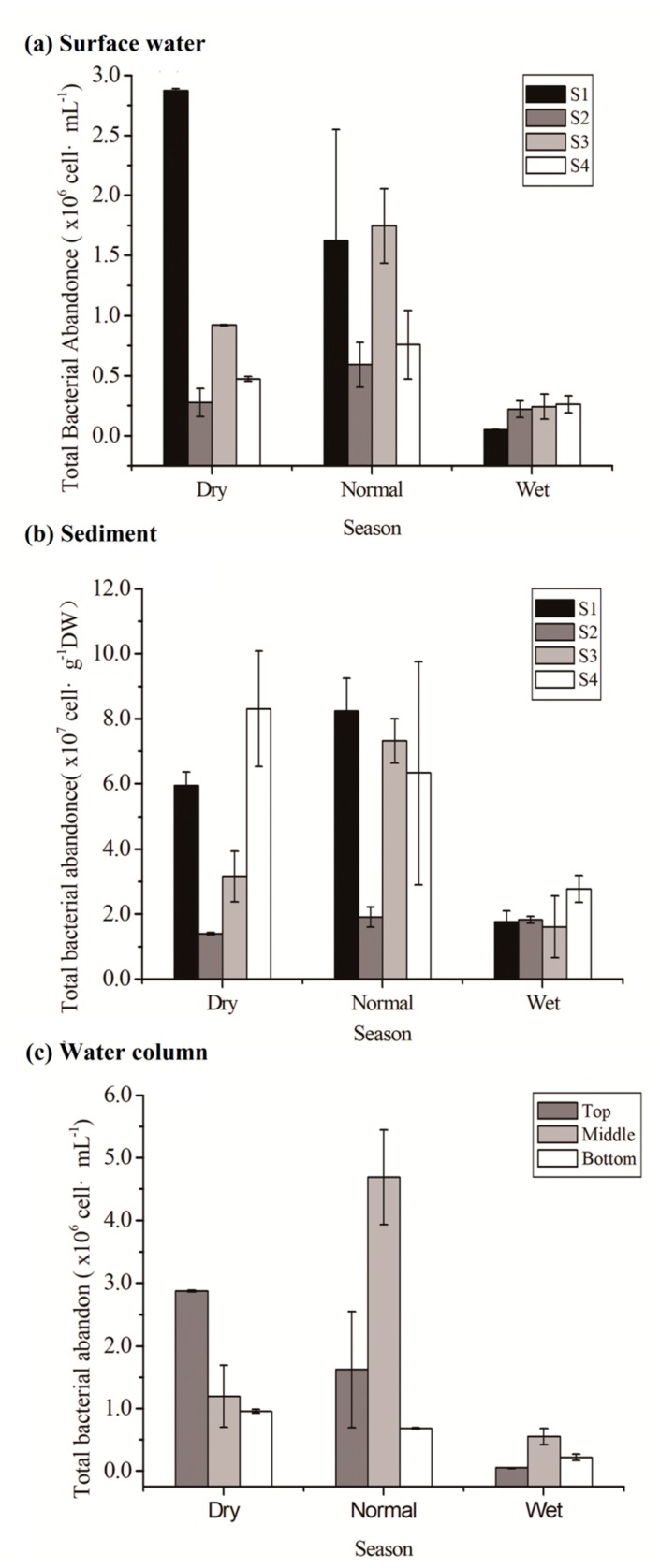
Total bacterial abundance in surface water (**a**), sediment (**b**), and water column (S1) (**c**) measured during different seasons. Error bars were calculated considering an average value derived from triplicate determinations carried out for each site. T: top; M: middle; B: bottom.

Bacterial abundance in surface water differed significantly from that in sediment (paired *t*-test, *p* < 0.01), being highest in the wet season, followed by those in normal and dry seasons. Compared to those in water, bacterial counts in sediment were 5–377 times higher (*p* < 0.01) (Table 2). In water, the bacterial abundance differed significantly between sites (*p* < 0.01) in the dry season. The largest differences were between S1 and those downstream of this location (Figure 3a). In all three seasons, the lowest bacterial abundance values in water down the stream were found immediately below the Gongguoqiao Reservoir (S2). Further downstream, the bacterial abundance was at or higher than the levels of recorded upstream in normal and rainy seasons. The highest bacterial density in water, nearly twofold to tenfold higher than the other values, was found in the reservoir in the dry season. Similarly, there were significant differences in the bacterial abundance in sediment according to the location (*p* < 0.01) in the dry season. The lowest bacterial abundance was recorded from samples taken immediately below the reservoir (Figure 3b). Beyond this point, bacterial abundance increased. 

When bacterial abundance in the reservoir was examined as a function of water depth, no depth dependency was found (Figure 3c): in normal and wet seasons, the highest value was obtained at the middle layer, whereas in the dry season, the highest value was observed at the top layer. 

**Figure 4:**
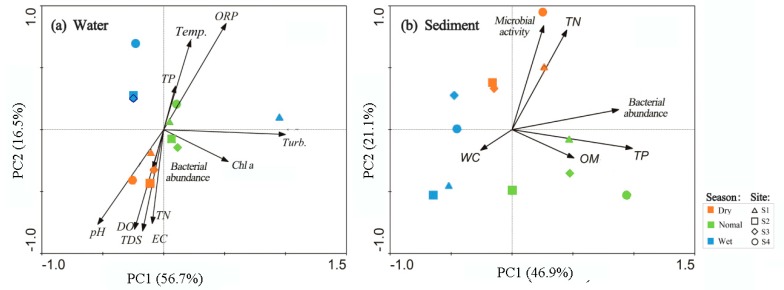
Principal component analysis (PCA) plot of physico-chemical variables in water (**a**) and sediment (**b**) of the sampling sites. EC: electric conductivity; Turd.: turbidity; Temp.: water temperature; WC: water content; OM: organic matter; ORP: oxidation–reduction potential.

That is, bacterial counts were consistently low in water and sediment during the rainy season. Searching for other site-based differences in bacterial abundance data (e.g., season sampled) yielded a significant determinant (Table 3). These results were reinforced by PCA (Figure 4). Score plots of PCA showed the water and sediment samples were clustered according to seasonal changes, whereas each of the sampling sites (S1–S4) was not well separated from the others.

#### 3.2.2. Algae biomass (Chl a)

Chl a in water was generally less than 2 µg/L, with concentrations rising in the reservoir generally to 2.2–3.3 µg/L during dry and wet seasons and decreasing in the downstream (Table 1). These observed treads are corroborated by the analysis of variance, which found a significant (*p* < 0.01) main effect for distance, with S1 having higher algae biomass than downstream sites during dry and wet seasons. At S1, the difference between surface and bottom algae biomass reached more than 5 µg/L in the wet season (see Table S5 in Supplemental Materials). The deeper water above the dam, however, did not seem to influence the algae biomass along the water column in the normal season (*p* > 0.05).

### 3.3. Relations Between Bacterial Abundance and Environmental Parameters

**Figure 5:**
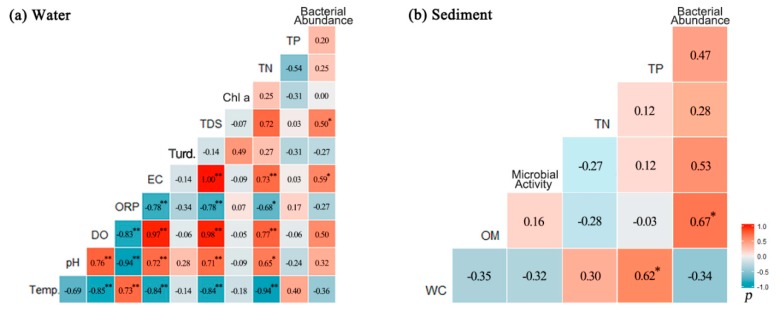
Pearson’s correlation coefficient among environmental parameters in water (**a**) and sediment (**b**). * *p*-value < 0.05, ** *p*-value < 0.01. EC: electric conductivity; Turd.: turbidity; Temp.: water temperature; WC: water content; OM: organic matter; ORP: oxidation–reduction potential.

Correlations between environmental parameters and bacterial abundances in sediment and water were tested by Pearson’s correlation analyses (Figure 5). Specifically, the results indicated that the bacterial density in the water was positively correlated with conductivity (r = 0.59, *p* < 0.01) and TDSs (r = 0.62, *p* < 0.01). The positive correlations for TDS, EC, and bacterial abundance were further demonstrated by the PCA plots (Figure 4a). In contrast, the bacterial density in sediment showed a significant positive correlation with OM content (*r* = 0.67, *p* < 0.05), suggesting the OM may have been the dominant carbon source for bacteria in sediment. Bacterial abundance showed no significant relationship with algae biomass in water, with microbial activity in sediment, and with TP and TN in both water and sediment samples. Compared with the use of PCA, it reduced the environmental variables to 3 PCs that explain 82.7% of the variance of the original data set. Temperature, DO, TN, EC, and TDSs explained 16.5% (PC 2) of the total variation, while PC 3 (TP) accounted for 9.5% of the total variance. Directions of the DO, bacterial abundance, pH, TDSs, EC, and TN vectors indicated a more close correlation between dry-season samples and those variables. Similarly, for sediment samples, the dry-season samples, with a positive score along PC 2, were positioned on the top of the score plot, while the normal-season samples, with a positive score along PC 1, were well separated from dry-season and wet-season samples that showed a negative score, as indicated in Figure 4b. PC 1 (47%), including bacterial abundance, OM, and TP, and PC 2 (21.1%), including microbial activity and TN, accounted for 68% of the total variance. More specifically, dry-season samples exhibited a higher concentration of TN and microbial activities. Normal-season samples were associated with OM content and TP, while wet-season samples were more closely associated with the sediment WC.

## 4. Discussion

Our results showed that bacterial abundances in both water and sediment of dammed river had a strong spatio-temporal dynamic. Physico-chemical differences between seasons, such as EC, TN, TP, and DO, were related to water release and precipitations. There was a large seasonal variation in bacterial abundance in the Gongguoqiao hydroelectric dam, which likely affected a broad array of physiological and geochemical estuarine processes. Bacterial density reduced significantly in the wet season. Relative to dry and normal seasons, seasonal precipitation and significant physical dilution of surface water due to river runoff would be the major cause for seasonal variations in bacterial abundance. One possible explanation for a lower bacterial density in the wet season was that the sewage treatment plant and agriculture land were far away from the dam reservoir, which was likely to provide an effective means to remove microbial pollutants before they reach the reservoir. In this case, the dilution effect due to rainfall became much more significant as seen in previous studies [39,40]. Bacterial density reduction potential in the normal season and wet season may be greatly enhanced by discharging water into reservoir and downstream of the dam in the form of heavy rainfall runoff rather than as a lower continuous flow. Moreover, higher densities of protozoan and metazoan grazer in the rainy season can decrease bacterial abundance through increasing bacterial mortality by direct grazing [41] and through decreasing nutrient concentration by consumption [42]. However, this finding contradicts the results of previous studies in the South Nation River Basin in Eastern Ontario, Canada [43] and in the Göta Älv River, Sweden [44], which reported that additional loading from non-point-source runoff, as well as from river bottom resuspension, were responsible for the increase in indicator bacteria densities and pathogen detection after rainfall.

In addition to the temporal variations in the bacterial density of Gongguoqiao hydroelectric dam, spatial distribution of the bacterial density was also explored in this study. The results suggested that the lowest bacterial abundance during the wet season was found in the reservoir (i.e., S1). Whereas the lowest bacterial abundance was found at S2 in normal and dry seasons. Similarly, significantly lower indicator bacterial counts downstream of reservoirs have been previously reported by Gannon et al. [45]. They found that the low flow velocities and residence times within reservoirs were significant causes of bacterial die-off and bacterial sedimentation. Moreover, the abundance and distribution of suspended particles can offer clues to this phenomenon. Often, bacteria that are attached to suspended particles consist of a significant proportion of the total bacteria in aquatic environments [46]. In normal and dry seasons, flow reduction below the dam and the accumulation and sedimentation of bacteria associated with particles in the reservoir [47] reduced the bacterial abundance significantly at S2. In dry and normal seasons, a large volume of sediment was trapped behind the dam. However, in wet seasons, this sediment was released into downstream, which caused the spiking of suspended particles and subsequently increased the bacterial abundance as observed at S2. 

In contrast, the bacterial abundance in sediments was always lowest at the site immediately below the dam (i.e., S2). We speculate that this may have been due to the particle size. Sediments at S2, which consistently supported the lowest concentration of bacterial cells, had a much coarse particle size distributions. These results were consistent with studies performed on another hydroelectric dam called Manwan in the Lancang River [23,48]. Both Liu et al. [48] and Zhao et al. [23] found that sand and micro-sand fractions become the major fraction in the sediments downstream of Manwan Dam, and the mean sizes of downstream sediments are obviously higher than the upstream. Under the influence of a dam, fine suspended particles are captured and accumulated from the floodplain, while coarse sediments become dominant due to the erosion of the downstream channel [24,26,49]. Previous work showed that particle size has a negative impact on microbial biomass density [50,51], and thus, an increased sediment particle size below the dam resulting from fast flowing water may allow few bacteria to attach to sediment particles, which decreases the density of bacterial cells in sediments at S2. Decamp and Warren [52] and Davies and Bavor [53] found that fine-texture sediments provide protection for bacteria from predators. Another reason for the higher density of bacterial cell in sediments with predominantly fine particles could be participation of bacteria in biofilm formation [54]. Garzio-Hadzick et al. [54] demonstrated that clay particles are shown to be conductive for the formation of biofilms in sediments.

During the analysis of the data, we were initially surprised to find that bacterial abundances were not associated with nutrients concentrations, such as TN and TP. It was known that bacterial abundance might be affected by OM, phosphorus, and nitrogen [5,6]. Retention in reservoirs can greatly reduce the delivery of N and P to downstream areas [16,55], influencing regional nutrient limitation patterns. However, in this case, the expansion of farmland, fertilizer use, and wastewater effluent from populated villages nearby delivered relatively more TP and TN to the downstream of the reservoir than natural sources, and thus bacterial abundance may be more limited by other factors when nutrients are abundant in water. Bacterial abundances in water were positively linked with the physicochemical characteristics of the water, such as TDSs and EC. These factors have been described previously to drive the formation of densely packed microbial cell communities termed biofilms [56]. He et al. [56] observed cations in solutions can promote the formation of surface biofilms by regulating protein expression and polysaccharide synthesis in extracellular polymeric substance produced by bacterial cells, resulting in higher total bacterial counts. Therefore, higher TDSs and EC would facilitate the growth of biofilm on the surface of suspended particles, leading to an increase in bacterial counts in water. This trend was in accordance with the findings in the Neuse River Estuary, North Carolina by Fries et al. [57], who reported that concentrations of *Escherichia coli* are strongly correlated with salinity (r = 0.72, *p* < 0.01). However, differently, a negative relationship between EC and bacterial density was observed by He et al. [58]. They reported that high levels of conductivity in the surface water stream of the San Diego region in Southern California provide high salt concentration which inhibit bacterial growth or even damage microorganisms. We acknowledge, however, that the data set may not be large enough or provide a wide enough span of conductivity values for the influence of some of the measured characteristics to be detected. For sediment samples, bacterial density in sediment was closely linked to OM. River sediment acts as a reservoir for bacteria [59,60,61], and primary production of heterotrophic bacteria tend to be limited by P concentrations and/or high N:P ratios [62]. However, P pools are accumulated and recycled in surface sediments due to P domestic inputs from direct wastewater release, so that sediments in reservoir and downstream of the dam are not expected to display P-limited conditions. Alternatively, slow-release and polymeric nutrients which are contained in higher OM sediments could retard cell die-off as reported in other studies [54]. 

To examine the relative contributions of bacterial abundance in sediment to the bacterial density in water, bacterial abundances in the top, middle, and bottom layers of the water column at the reservoir (i.e., S1) were also measured. Bacterial abundance was found to be likely affected by water temperature in the dry season. Gurung and Urabe [42] attributed a positive correlation between bacterial biomass and temperature to the bacteria growth rate when resources such as phosphorus are sufficient. Quinn et al. [63] also pointed out that the inhibited bacterial activities and growth rate in cold, deep layers may result in the decrease in bacterial biomass. Conversely, as precipitation and riverine discharge increased during the normal season and wet season, there were higher turbidity and a decrease in water residence time, allowing for the buildup of bacterial cells in the middle layer of S1. Clearly, hydropower-related discharge often occurred in the middle layer of water column, the increased bacterial abundance at this layer was also likely supported by an increase in flood-induced turbidity [64] since a large amount of particulate nutrients and sediments originated from heavy rainfall runoff could affect bacterial abundance in the water column [65]. 

The difference of bacterial abundance between surface water and sediment was highest in the wet season, followed by those in the normal season and dry season. As our sampling in the rainy season occurring after a significant high flow event, high flow velocity [66], together with the significant physical dilution of surface water, due to river runoff most likely, caused a drop of mixing and may explain the significant difference between bacterial biomass in sediment and surface water. A significant difference in bacterial abundance between overlying water and sediment in the reservoir was noticed in the normal season and wet season. It has been reported that alternating high and low oxygen waters would induce significant water–sediment fluxes of dissolved phosphorus [57] and possibly promote bacteria persistence or proliferation under hypoxic conditions. While as mentioned above, hydropower-related discharge at the Gongguoqiao hydroelectric dam often occurred in the middle layer of the water column, the high sediment concentrations at the middle layer, instead of those at the bottom layer, were likely to have provided the source of these cells. 

## 5. Conclusions

This study assessed the abundance of bacteria, physicochemical characteristics, algae biomass of water and sediment, and their interactions in the presence of hydroelectric dam. The following conclusions are drawn:

(1). The presence of dam greatly modified important habitat conditions such as DO, EC, and turbidity in water, as well as WC, TN, and TP in sediment.
(2). Although the retention of P and N in the reservoir led to the nutrient reduction in the downstream of Lancang River, bacterial density continued to grow as anthropogenic sources delivered more nutrients to the downstream river than natural sources.
(3). The effects of hydropower discharge and strong precipitation would be major causes for seasonal variations in bacterial abundance and physicochemical characteristics. Water discharge variations and the enhanced precipitation in the wet season also promoted significant differences in the conditions of the river below the dam, such as the concentration of DO, ORP, EC, turbidity, and TDSs in water and concentrations of microbial activity in sediment.
(4). Bacterial abundance was highest in the reservoir during the dry season and decreased during normal and wet seasons. Physicochemical characteristics of the water, such as TDSs and EC, explained a greater proportion of the variations in bacterial abundance in water. In contrast, bacterial density in sediment was related with hydropower-related discharge, particle size, and type of sediments.
(5). Flow release did influence the stratification in the dammed reservoir so that stratified conditions were not observed in normal and wet seasons. Higher turbidity and a decrease in water residence time during normal and wet seasons resulted in the buildup of bacterial cells in the middle layer of the reservoir, whereas during the dry season, bacterial density in the reservoir was affected by water temperature.

Our study provides baseline information for predicting regional-scale responses to anthropogenic changes. It also highlights the ecological importance of bacterial density for the proper assessment of microbial pollution of the overlying water and the management of the trophic state of freshwater. However, this study only focused on the general changes of bacterial abundance and physicochemical characteristics in sediment and water, and a major aspect missing in this study is information on the bacterial community structure, function of bacterial assemblages, and their potential responses to dam construction. Future work is necessary in order to determine what fraction of the microbial community is active in the water column and sediment from season to season, and to elucidate the links between environmental factors and microbial community in the presence of hydroelectric dam. 

## Figures and Tables

**Table 1:** Physical and chemical characteristics (mean ± standard deviation) in water upstream and downstream of Gongguoqiao hydroelectric dam.

Site	Temperature (°C)	pH	DO (mg·L^−1^)	ORP (mV)	EC (μS·cm^−1^)	Turbidity (NTU)	Chl a (μg·L^−1^)	TDS (g·L^−1^)	TN (mg·L^−1^)	TP (mg·L^−1^)	Bacterial Abundance (×10^6^ Cell·mL^−1^)
Dry season											
S1	10.9 ± 0.2	8.0 ± 0.2	15.9 ± 1.3	240.7 ± 5.8	48.6 ± 2.5	84.9 ± 14.4	2.2 ^1^	0.3 ± 0.0	1.6 ± 0.1	0.02 ± 0.00	2.87 ± 0.01
S2	11.6 ± 1.2	8.0 ± 0.1	16.9 ± 2.9	207.0 ± 11.4	49.1 ± 1.2	85.5 ± 2.2	1.3 ^1^	0.3 ± 0.0	1.5 ± 0.1	0.03 ± 0.01	0.28 ± 0.12
S3	10.2 ± 0.6	8.1 ± 0.0	14.2 ± 0.5	224.7 ± 2.5	51.1 ± 0.6	99.4 ± 9.5	1.4 ^1^	0.3 ± 0.0	1.7± 0.0	0.03 ± 0.00	0.92 ± 0.01
S4	13.1 ± 0.1	8.2 ± 0.2	11.4 ± 1.4	197.3 ± 11.6	41.8 ± 0.2	10,300.0 ± 667.9	2.9 ^1^	0.3 ± 0.0	1.5 ± 0.1	0.10 ± 0.02	0.47 ± 0.02
Normal season											
S1	18.0 ± 0.5	7.6 ± 0.0	11.4 ± 1.4	284.7 ± 10.2	40.5 ± 0.2	158.7 ± 0.6	1.3 ± 0.1	0.3 ± 0.0	1.5 ± 0.3	0.03 ± 0.06	1.62 ± 0.93
S2	21.0 ± 0.1	7.4 ± 0.1	7.4 ± 0.6	267.7 ± 27.0	41.9 ± 0.1	170.3 ± 3.2	1.2 ± 0.0	0.3 ± 0.0	1.3 ± 0.3	0.14 ± 0.08	0.59 ± 0.19
S3	17.9 ± 0.8	7.3 ± 0.0	8.2 ± 0.4	262.3 ± 37.5	41.1 ± 0.3	197.3 ± 19.3	1.5 ± 0.1	0.3 ± 0.0	1.4 ± 0.1	0.16 ± 0.05	1.74 ± 0.31
S4	17.4 ± 0.6	7.2 ± 0.1	8.5 ± 0.2	309.3 ± 8.1	40.8 ± 0.2	185.3 ± 1.5	1.3 ± 0.0	0.3 ± 0.0	1.5 ± 0.2	0.85 ± 0.13	0.76 ± 0.29
Wet season											
S1	19.1 ± 0.5	6.5 ± 0.0	4.0 ± 0.1	360.0 ± 0.0	32.1 ± 0.3	625.7 ± 149.6	3.3 ± 0.3	0.2 ± 0.0	0.4 ± 0.1	0.10 ± 0.02	0.05 ± 0.01
S2	20.3 ± 2.1	7.5 ± 0.1	4.1 ± 0.4	289.0 ± 6.6	32.5 ± 0.4	1715.0 ± 17.3	1.4 ± 0.1	0.2 ± 0.0	0.3 ± 0.0	0.10 ± 0.08	0.22 ± 0.07
S3	20.0 ± 1.9	7.7 ± 0.0	5.4 ± 0.2	286.0 ± 0.0	31.8 ± 0.8	1826.7 ± 86.7	1.2 ± 0.0	0.2 ± 0.0	0.4 ± 0.2	0.06 ± 0.01	0.24 ± 0.01
S4	19.2 ± 0.6	6.4 ± 0.0	3.3 ± 0.1	347.3 ± 1.5	31.1 ± 0.3	1815.6 ± 10.2	1.3 ± 0.0	0.2 ± 0.0	0.3 ± 0.1	0.24 ± 0.01	0.26 ± 0.07

^1^ Only one sample was measured.

**Table 2:** Physical and chemical characteristics (mean ± standard deviation) in sediment upstream and downstream of Gongguoqiao hydroelectric dam.

Site	WC (%)	OM (%)	TN (mg·g^−1^ DW)	TP (mg·g^−1^ DW)	Microbial Activity (µg FDA g^−1^ DW h^−1^)	Bacterial Abundance (×10^7^ cell·g^−1^ DW)
Dry season						
S1	50.8 ± 4.0	2.7 ± 3.5	5.4 ± 0.3	4.3 ± 1.2	2.5 ± 0.0	5.94 ± 0.42
S2	79.4 ± 4.6	0.8 ± 0.2	4.8 ± 0.2	2.5 ± 0.1	2.0 ± 0.1	1.40 ± 0.04
S3	69.0 ± 3.6	1.4 ± 0.3	5.0 ± 0.2	2.5 ± 0.1	1.6 ± 0.3	3.16 ± 0.78
S4	65.8 ± 0.4	3.6 ± 2.0	5.7 ± 0.1	3.9 ± 0.1	3.8 ± 0.9	8.30 ± 1.780
Normal season						
S1	55.2 ± 8.7	13.0 ± 2.1	4.3 ± 0.1	5.77 ± 0.6	1.7 ± 0.2	8.24 ± 1.00
S2	79.2 ± 6.8	2.6 ± 0.5	3.5 ± 0.1	3.8 ± 0.3	0.3 ± 0.0	1.91 ± 0.31
S3	64.9 ± 5.2	4.9 ± 0.6	4.2 ± 0.2	5.9 ± 0.3	0.8 ± 0.3	7.31 ± 0.68
S4	68.0 ± 3.8	2.6 ± 0.0	4.5 ± 0.4	8.2 ± 0.0	0.6 ± 0.1	6.33 ± 3.43
Wet season						
S1	56.1 ± 4.0	2.2 ± 0.2	2.4 ± 3.0	0.9 ± 0.0	0.6 ± 0.1	1.76 ± 0.34
S2	79.8 ± 0.9	1.2 ± 0.1	1.7 ± 1.4	0.5 ± 0.0	0.8 ± 0.3	1.82 ± 0.1
S3	76.8 ± 0.1	0.8 ± 0.0	5.2 ± 0.0	0.9± 0.2	0.6 ± 0.2	1.61 ± 0.95
S4	66.9 ± 0.2	1.5 ± 0.4	4.1 ± 0.5	1.2 ± 0.2	0.6 ± 0.2	2.77 ± 0.41

**Table 3:** Effects of site (S1, S2, S3, and S4) and sampling season (dry, normal, and wet) on the physicochemical characteristics of water and sediment by means of repeated measures ANOVA. NS: no significant effect at *p* = 0.05.

Water							Sediment						
	Site (S)		Sampling Season (Ss)		S × Ss			Site (S)		Sampling Season (Ss)		S × Ss	
	F	*p*	F	*p*	F	*p*		F	*p*	F	*p*	F	*p*
Temperature (°C)	5.55	0.007 ^NS^	10.25	<0.01	4.36	0.043	WC (%)	96.03	<0.01	12.12	<0.01	2.38	0.110 ^NS^
pH	2.66	0.154 ^NS^	137.81	<0.01	110.38	0.011	OM (%)	68.92	<0.01	59.24	<0.01	29.99	<0.01
DO (mg·L^−1^)	20.59	<0.01	96.55	<0.01	16.56	<0.01	TN (mg·g^−1^ DW)	12.91	<0.01	7.74	0.022	8.43	0.002
ORP (mV)	15.36	<0.01	178.22	<0.01	7.06	<0.01	TP (mg·g^−1^ DW)	36.73	<0.01	477.05	<0.01	14.44	<0.01
EC (μS·cm^−1^)	39.67	<0.01	550.78	<0.01	24.67	<0.01	Microbial activity (µg FDA·g^−1^ DW·h^−1^)	68.05	<0.01	168.52	<0.01	35.62	<0.01
Turbidity (NTU)	675.66	<0.01	494.93	<0.01	657.13	<0.01	Bacterial abundance (×10^7^ cell·g^−1^ DW)	26.12	<0.01	44.64	<0.01	7.99	<0.01
Chl a (μg·L^−1^)	233.17	<0.01	170.16	<0.01	196.04	<0.01							
TDS (g·L^−1^)	129.27	<0.01	1962.18	<0.01	83.94	<0.01							
TN (mg·L^−1^)	1.60	<0.01	307.07	<0.01	0.57	<0.01							
TP (mg·L^−1^)	84.01	<0.01	85.88	<0.01	28.84	<0.01							
Bacterial abundance (×10^6^ cell·mL^−1^)	21.55	<0.01	90.72	<0.01	14.07	<0.01							

## Supplementary Materials

The following are available online at https://www.mdpi.com/1660-4601/16/11/2031/s1, Table S1: Physical and chemical characteristics and ANOVA (F and *p*) at different depths in Gongguoqiao Reservoir.
