# Supplementary material for: Bacterial Abundance and Physicochemical Characteristics of Water and Sediment Associated with Hydroelectric Dam on the Lancang River China
Source: Int J Environ Res Public Health. 2019 Jun 7;16(11):2031. doi: 10.3390/ijerph16112031 (PMC6603985; doi:10.3390/ijerph16112031)
Supplement: Supplementary file 1 [file ijerph-16-02031-s001.pdf]

Table S1. Summary of Gongguoqiao hydroelectric dam operation condition and rainfall distribution

Season	Outflow at the time of sampling ($m^3 \cdot s^{-1}$)	Mean head-water level (m)	Mean reservoir water level (m)	Reservoir storage capacity ($10^4 \cdot m^{-3}$)	Cumulative water flow ($10^4 \cdot m^{-3}$)					Mean 7-day rainfall before sampling (mm)
					Inflow	Storage	Outflow	Power generation	Discard water	
Dry	267	61.5	1303.8	27248	2565	581	1984	1984	0	0
Normal	524.18	60.54	1304	27237	5496	268	5228	5228	0	11.2
Wet	2268.33	56.08	1305.3	27400	19597	1519	17078	12165	4915	17.8

Table S2. Summary of sampling depth, time and number of samples taken on each research site

	Season (Time)	Site	Depth (m)	No. of samples taken
Water	Dry (January, 2017)	S1	0.2	3
			18.5	3
			37.5	3
		S2	0.2	3
		S3	0.2	3
		S4	0.2	3
	Normal (May, 2017)	S1	0.2	3
			21.0	3
			42.5	3
		S2	0.2	3
		S3	0.2	3
		S4	0.2	3
	Wet (July, 2017)	S1	0.2	3
			7.0	3
			14.2	3
		S2	0.2	3
		S3	0.2	3
		S4	0.2	3
Sediment	Dry (January, 2017)	S1	0.1	3
		S2	0.1	3
		S3	0.1	3
		S4	0.1	3
	Normal (May, 2017)	S1	0.1	3
		S2	0.1	3
		S3	0.1	3
		S4	0.1	3
	Wet (July, 2017)	S1	0.1	3
		S2	0.1	3
		S3	0.1	3
		S4	0.1	3

Table S3. Raw data of physical and chemical characteristics in water upstream and downstream of Gongguoqiao hydroelectric dam

Site	Temperature (°C)	pH	DO (mg·L^{-1})	ORP (mV)	EC (μS·cm^{-1})	Turbidity (NTU)	Chl a (μg·L^{-1})	TDS (g·L^{-1})	TN (mg·L^{-1})	TP (mg·L^{-1})	Bacterial abundance (×10^{6} cell·mL^{-1})
Dry season											
S1①	10.88	8.15	16.92	244	48.3	92.4	2.2[1]	0.320	1.476	0.026	2.88
S1②	10.83	7.7	16.17	244	46.30	94.1		0.320	1.617	0.022	2.86
S1③	11.22	8.23	14.56	234	51.3	68.4		0.328	1.546	0.024	2.87
S2①	12.99	8.05	19.92	199	48.3	85.9	1.3[1]	0.314	1.420	0.026	0.40
S2②	11.4	7.89	17.01	202	48.50	87.5		0.314	1.575	0.042	0.16
S2③	10.59	8.09	14.06	220	50.5	83.2		0.323	1.497	0.034	0.28
S3①	10.99	8.1	14.75	222	50.5	110.0	1.4[1]	0.323	1.715	0.026	0.93
S3②	10.1	8.13	14.31	225	51.20	96.6		0.328	1.673	0.026	0.91
S3③	9.75	8.15	13.67	227	51.6	91.6		0.330	1.694	0.026	0.92
S4①	12.97	8.4	12.94	199	42	9645.0	2.9[1]	0.273	1.490	0.102	0.47
S4②	13.25	8.03	10.95	185	41.80	10980.0		0.272	1.589	0.115	0.49
S4③	13.11	8.15	10.23	208	41.7	10275.0		0.271	1.392	0.088	0.45
Normal season											
S1①	18.64	7.64	12.93	273	40.3	158.0	1.19	0.262	1.318	0.059	2.54
S1②	17.49	7.57	11.09	289	40.60	159.0	1.34	0.264	1.864	0.059	0.70
S1③	17.89	7.55	10.13	292	40.7	159.0	1.19	0.264	1.341	0.059	1.62
S2①	20.91	7.46	7.99	241	42.1	168.0	1.18	0.274	1.376	0.158	0.59
S2②	21.06	7.38	7.47	267	41.90	169.0	1.17	0.273	1.028	0.059	0.77
S2③	21.15	7.35	6.8	295	41.9	174.0	1.22	0.273	1.516	0.207	0.40
S3①	18.96	7.3	8.31	283	41	182.0	1.48	0.267	1.446	0.108	1.74
S3②	17.62	7.3	8.47	219	40.80	219.0	1.51	0.265	1.446	0.207	2.06
S3③	17.31	7.34	7.67	285	41.4	191.0	1.57	0.269	1.341	0.158	1.43
S4①	18.09	7.07	8.33	313	40.6	184.0	1.32	0.264	1.423	0.700	0.76
S4②	17.47	7.08	8.48	315	40.90	185.0	1.38	0.266	1.376	0.946	1.04
S4③	16.75	7.31	8.72	300	40.9	187.0	1.36	0.266	1.702	0.897	0.47
Wet season											
S1①	19.77	6.52	4.01	360	31.8	794	3.09	0.207	0.306	0.083	0.05

S1②	18.79	6.49	4.02	360	32.2	575	3.58	0.209	0.425	0.113	0.05
S1③	18.91	6.5	3.91	360	32.3	508	3.15	0.21	0.575	0.098	0.04
S2①	20.62	7.43	3.88	290	32.6	1725	1.35	0.211	0.246	0.053	0.22
S2②	18.08	7.65	3.91	282	32.8	1695	1.37	0.213	0.261	0.193	0.29
S2③	22.36	7.56	4.56	295	32	1725	1.47	0.208	0.246	0.049	0.15
S3①	21.9	7.66	5.37	286	31.2	2775	1.26	0.203	0.455	0.071	0.24
S3②	20.1	7.66	5.58	286	31.6	2962.5	1.22	0.205	0.142	0.056	0.14
S3③	18.02	7.64	5.29	286	32.7	2857.5	1.24	0.213	0.560	0.064	0.35
S4①	19.99	6.39	3.21	347	30.7	1813.3	1.31	0.200	0.276	0.245	0.26
S4②	18.95	6.44	3.33	346	31.2	1826.7	1.37	0.203	0.276	0.234	0.20
S4③	18.72	6.46	3.34	349	31.3	1806.7	1.28	0.204	0.440	0.240	0.33

[1]Only one sample was measured.

Table S4. Raw data of physical and chemical characteristics in sediment upstream and downstream of Gongguoqiao hydroelectric dam[1]

Site	WC (%)	OM (%)	TN ($mg·gDW^{-1}$)	TP ($mg·gDW^{-1}$)	Microbial activity (μg FDA$·g^{-1}$ DW $·h^{-1}$)	Bacterial abundance ($\times 10^7$ cell$·gDW^{-1}$)
Dry season						
S1①	53.70	5.20	5.59	3.52	2.544	5.94
S1②	47.98	0.28	5.13	5.14	2.544	6.36
S1③	50.84	2.74	5.36	4.33	2.544	5.53
S2①	76.16	0.95	5.01	2.55	2.022	1.40
S2②	82.72	0.73	4.67	2.45	1.930	1.43
S2③	79.44	0.84	4.84	2.50	1.976	1.36
S3①	66.45	1.62	5.15	2.45	1.762	3.16
S3②	71.49	1.26	4.83	2.56	1.375	2.38
S3③	68.97	1.44	4.99	2.50	1.568	3.94
S4①	66.02	5.08	5.80	3.97	3.166	8.30
S4②	65.51	2.21	5.70	3.88	4.481	10.08
S4③	65.77	3.64	5.75	3.92	3.823	6.52
Normal season						
S1①	49.06	11.50	4.23	5.23	1.578	8.24
S1②	61.31	14.43	4.36	6.08	1.839	7.23
S1③	55.18	12.96	4.29	5.66	1.708	9.24
S2①	84.03	2.22	3.52	3.65	0.356	1.91
S2②	74.37	2.94	3.39	4.05	0.283	1.60
S2③	79.20	2.58	3.45	3.85	0.319	2.21
S3①	61.17	4.50	4.29	6.08	1.037	7.31
S3②	68.54	5.38	4.08	5.66	0.557	8.00
S3③	64.86	4.94	4.19	5.87	0.797	6.64
S4①	65.28	2.62	4.78	8.18	0.716	6.33
S4②	70.71	2.62	4.20	8.25	0.540	9.76

S4③	68.00	2.62	4.49	8.22	0.628	2.90
Wet season						
S1①	58.97	2.08	4.51	0.91	0.531	1.76
S1②	53.28	2.35	0.31	0.87	0.656	1.42
S1③	56.12	2.22	2.41	0.89	0.593	2.10
S2①	80.46	1.07	2.73	0.53	0.643	1.82
S2②	79.17	1.26	0.73	0.53	1.014	1.92
S2③	79.82	1.16	1.73	0.53	0.828	1.72
S3①	76.74	0.82	5.19	0.55	0.397	1.61
S3②	76.88	0.88	5.13	0.71	0.731	0.66
S3③	76.81	0.85	5.16	0.63	0.564	2.56
S4①	67.10	1.79	3.80	0.71	0.805	2.77
S4②	66.76	1.27	4.46	0.93	0.455	2.36
S4③	66.93	1.53	4.13	0.82	0.630	3.19

Table S5. Physical and chemical characteristics at different depths in Gongguoqiao reservoir[1, 2]

Site	Temperature (℃)	pH	DO ($mg·L^{-1}$)	ORP (mV)	EC ($\mu S·cm^{-1}$)	Turbidity (NTU)	Algae Biomass ($\mu g·L^{-1}$)	TDS ($g·L^{-1}$)	TN ($mg·L^{-1}$)	TP ($mg·L^{-1}$)	Bacterial abundance ($\times 10^6$ $cell·mL^{-1}$)
Dry season											
S1T	11.0±0.2	8.0±0.2	15.9±1.0	240.7±4.7	48.6±2.1	84.9±11.7	2.3±0.1	0.3±0.0	1.5±0.1	0.02±0.00	2.87±0.01
S1M	9.73±0.5	7.7±0.3	14.7±0.1	245.0±0.0	49.9±1.0	87.2±2.3	1.7±0.0	0.3±0.0	1.3±0.1	0.02±0.01	0.55±0.11
S1B	9.52±0.2	7.0±0.0	13.7±0.2	234.0±0.0	51.3±0.5	88.4±3.6	1.5±0.0	0.3±0.0	1.3±0.1	0.01±0.00	0.22±0.04
Normal season											
S1T	18.0±0.5	7.6±0.0	11.4±1.2	284.7±8.3	40.5±0.2	158.7±0.5	1.2±0.3	0.3±0.0	1.5±0.3	0.06±0.00	1.62±0.76
S1M	16.2±0.1	7.6±0.0	10.4±0.4	304.7±3.7	42.0±0.5	173.7±1.2	0.8±0.0	0.3±0.0	0.7±0.1	0.06±0.00	4.69±0.62
S1B	15.8±0.1	7.5±0.0	9.3±0.3	306.7±2.6	42.3±0.3	176.0±5.0	1.0±0.0	0.3±0.0	4.9±1.0	0.10±0.00	0.68±0.01
Wet season											
S1T	19.2±0.4	6.5±0.0	4.0±0.0	360.0±0.0	32.1±0.2	625.7±122.1	3.3±0.3	0.2±0.0	0.4±0.1	0.10±0.01	0.05±0.01
S1M	18.7±0.2	6.4±0.0	4.1±0.2	349.7±12.5	32.5±0.1	1,826.7±70.8	1.5±0.1	0.2±0.0	0.6±0.1	0.29±0.01	0.55±0.11
S1B	16.74±1.3	6.4±0.0	4.0±0.0	342.3±3.7	32.3±0.0	637±291.5	8.4±0.7	0.2±0.0	0.3±0.0	0.21±0.06	0.22±0.04

[1]Values are means ± standard deviation on mean, n=3

[2]S1T, S1M, and S1B represent the top, middle and bottom layer at the dam reservoir (i.e., site S1), respectively.

Table S6. Effects of water depth (top, middle and bottom) and sampling season (dry, normal and wet) on the physicochemical characteristics of water in the dam reservoir by means of repeated measures ANOVA. NS: no significant effect at p=0.05.

	Depth (D)	Sampling season (Ss)	D × Ss

	F	p	F	p	F	p
Temperature (°C)	52.05	< 0.01	830.93	< 0.01	5.08	0.012
pH	13.53	< 0.01	282.82	< 0.01	9.35	< 0.01
DO ($mg \cdot L^{-1}$)	14.43	< 0.01	352.70	< 0.01	3.60	0.038
ORP (mV)	1.79	0.209 ^{NS}	513.72	< 0.01	7.97	0.002
EC ($\mu S \cdot cm^{-1}$)	4.69	0.031	826.03	< 0.01	0.88	0.505 ^{NS}
Turbidity (NTU)	42.91	< 0.01	81.81	< 0.01	41.82	< 0.01
Chl a ($\mu g \cdot L^{-1}$)	420.94	< 0.01	417.32	< 0.01	463.58	< 0.01
TDS ($g \cdot L^{-1}$)	233.72	< 0.01	3292.01	< 0.01	140.76	< 0.01
TN ($mg \cdot L^{-1}$)	17.90	< 0.01	45.02	< 0.01	24.05	< 0.01
TP ($mg \cdot L^{-1}$)	12.33	< 0.01	103.50	< 0.01	12.72	< 0.01
Bacterial abundance ($\times 10^6$ $cell \cdot mL^{-1}$)	68.66	< 0.01	30.08	< 0.01	81.12	< 0.01
